# Periodontitis in patients with cirrhosis: a cross-sectional study

**DOI:** 10.1186/s12903-018-0487-5

**Published:** 2018-02-13

**Authors:** Lea Ladegaard Grønkjær, Palle Holmstrup, Søren Schou, Johanne Kongstad, Peter Jepsen, Hendrik Vilstrup

**Affiliations:** 1 Department of Hepatology and Gastroenterology, Aarhus University Hospital, Nørrebrogade 44, 8000 Aarhus C, Denmark; 2 Section of Periodontology, Microbiology, and Community Dentistry, Department of Odontology, Faculty of Health and Medical Sciences, University of Copenhagen, Copenhagen, Denmark; 3 Section for Oral Surgery, Department of Odontology, Faculty of Health and Medical Sciences, University of Copenhagen, Copenhagen, Denmark; 4 Department of Clinical Epidemiology, Aarhus University Hospital, Aarhus, Denmark

**Keywords:** Cirrhosis, Inflammation status, Nutritional status, Oral health, Periodontitis

## Abstract

**Background:**

Many patients with cirrhosis have poor oral health but little is known on periodontitis, and its clinical significance is largely unknown. This study aimed to examine the prevalence and predictors of periodontitis, and evaluate the association of periodontitis with nutritional and systemic inflammation status.

**Methods:**

145 patients with cirrhosis were consecutively enrolled. Clinical, oral examination of plaque, pocket depth, clinical attachment level, and bleeding on probing was performed. Patients were categorized as having no-or-mild, moderate, or severe periodontitis. Predictors of severe periodontitis and the association with nutritional and systemic inflammation status were analyzed using univariable and multivariable logistic regression analyses.

**Results:**

The large majority of patients had periodontitis, 46% of them severely and 39% moderately. Predictors of severe periodontitis included smoking (odds ratio (OR) 2.93, 95% confidence interval (CI) 1.29–6.63), brushing teeth twice daily (OR 0.30, 95% CI 0.11–0.79), and visiting the dentist annually (OR 3.51, 95% CI 1.22–10.81). Cirrhosis etiology or severity was not predictors of severe periodontitis. The patients with severe periodontitis had a higher nutritional risk score than patients with moderate, mild, or no periodontitis (3, interquartile range (IQR) 3–5 vs. 3, IQR 2–4, *P* = 0.02).

**Conclusions:**

Most cirrhosis patients had significant periodontitis, the severity of which was related to life style factors and was associated with higher nutrition risk score. Our results emphasize the need for further research to establish the effect of periodontitis on cirrhosis.

## Background

Periodontitis is an inflammatory disease of multifactorial etiology that affects the supporting tissues of the teeth and is characterized by deepening of periodontal pockets, connective tissue attachment loss and alveolar bone loss [1]. Untreated, periodontitis can result in discomfort, impaired mastication, pain, and eventual tooth loss [1, 2].

Oral health is generally poor in patients with cirrhosis and may lead to oral infections [3–6]. However, the prevalence and risk factors for periodontitis in cirrhosis are sparsely investigated. In addition, it has been reported that alcoholic cirrhosis and the severity of cirrhosis may contribute to a higher risk of periodontitis, but the relationship has not been substantiated and data are conflicting [7–10].

Malnutrition and infections are common and serious complications to cirrhosis leading to increased morbidity and mortality [11, 12]. Periodontitis itself may have adverse health effects and it has been associated with malnutrition and systemic inflammation activation in patients with cardiovascular disease, chronic kidney disease, and diabetes [13–15]. It has not yet been investigated whether periodontitis is similarly associated in cirrhosis.

The aim of the present study, therefore, was to examine the prevalence and identify predictors of periodontitis, and to evaluate the association of periodontitis with nutritional and systemic inflammation status in a cohort of patients with cirrhosis.

## Methods

### Study design

Between April 2013 and April 2015, eligible patients from the Department of Hepatology and Gastroenterology at Aarhus University Hospital were enrolled consecutively – regardless of disease etiology and level of disease severity. The patient cohort was partly the same as in our previous study [3].

Eligible patients were adult > 18 years men and woman with an established diagnosis of cirrhosis based on either liver biopsy and/or clinical, biochemical, and ultrasonic findings, who were able to give consent and to co-operate to an oral examination, and had two or more teeth were eligible candidates and invited to participate in the study. The study was conducted in accordance with the Declaration of Helsinki. The study was approved by The Central Denmark Region Committees on Health Research Ethics (No. 1–10–72-128-12). Written, informed consent was obtained from all participating patients.

### Oral examination

Full mouth dental chart was recorded and all teeth were examined at six sites per tooth. Plaque was registered as 1 for visible plaque, if necessary after using the probe across the tooth surface, and 0 for no plaque, modified from Silness and Löe [16]. Clinical probing depths (PD) were measured parallel to the longitudinal axis of the tooth from the free gingival margin to the bottom of the periodontal pocket, i.e. to the tip of the periodontal probe. Clinical attachment level (CAL) was defined as the distance from the cementoenamel junction (CEJ) to the tip of the periodontal probe. The distance from the free gingival margin to the CEJ was measured and CAL was calculated by subtracting this value from PD. When the gingival margin had receded and the CEJ was exposed, it was an indication of gingival recession and a negative value was recorded and added to PD. PD and CAL was measured in millimetre and recorded to the nearest millimetre. Bleeding on probing (BOP) was registered as 0 if no bleeding and 1 if bleeding occurred within 15 s after probing. Mean values were calculated for all variables for each patient.

Periodontitis was defined as either no-or-mild, moderate or severe, as defined by the working group of the Centre for Disease Control and Prevention (CDC) in collaboration with American Academy of Periodontology (AAP) [17] (Table 1).

**Table 1:** Clinical case definition by the CDC/AAP working group for use in population-based surveillance studies of periodontitis

Category	Clinical attachment level (CAL)	Probing pocket depth (PD)
Severe periodontitis	≥ 2 interdental sites with CAL ≥ 6 mm (not on same tooth) and	≥ 1 interdental site with PD ≥ 5 mm
Moderate periodontitis	≥ 2 interdental sites with CAL ≥ 4 mm (not on same tooth) or	≥ 2 interdental sites with PD ≥ 5 mm (not on same tooth)
No-or-mild periodontitis	Neither “moderate” nor “severe” periodontitis	

Three authorised dental hygienists were responsible for the oral examinations, and they were trained by an experienced clinical examiner in periodontitis from the Department of Odontology, Aarhus University, prior to the study start. This was done in order to improve reproducibility. Eleven percent of the patients had at least two quadrants of their periodontal measurement (i.e. probing depth and clinical attachment level) repeated by the same or another hygienist in order to assess intra- and inter-examiner variability. The reproducibility was assessed by calculation of Lin’s concordance correlation coefficient, and the degree of agreement was assessed according to the categories suggested by McBride [18]. The lower one-sided 95% confidence level and the concordance correlation coefficient ranged from 0.90 to 0.96 and 0.91 to 0.96 respectively. Thus, the degree of agreement was moderate to substantial [18].

### Data collection

Information on patients’ age, gender, cirrhosis etiology, cirrhosis severity, smoking status (no smoking, former smoker, currently smoking), alcohol status, burden of comorbidity (Charlson comobidity index) [19], and oral care habits (i.e. tooth brushing frequency and dental visits frequency) were asked or collected from the medical charts. Model of End-Stage Liver Disease score (MELD) and C-reactive protein (CRP) were collected from the routine blood samples on the same day as the oral examination.

The patients’ nutritional risk was assessed by the screening tool NRS-2002 [20]. Patients were scored in three domains: (a) nutrition status measured by body mass index and nutritional intake, (b) disease severity, and (c) age, giving a total score from 0 to7. A score of 3 or above defines high nutritional risk and need of targeted nutritional therapy. Handgrip strength was measured using a dynamometer and expressed in kg. The highest of three measurements on the dominant hand was used for analyses [21].

### Statistical analysis

Univariable and multivariable including stepwise logistic regression [22] analyses were performed to examine predictors associated with severe periodontitis as opposed to no-mild, or moderate periodontitis, and to evaluate the association between the presence of severe periodontitis and nutrition and systemic inflammation status.

The predictor variables were age, male gender (yes/no), alcoholic cirrhosis (yes/no), MELD score, smoking (yes/no), current alcohol use (yes/no), diabetes (yes/no), brush teeth twice daily (yes/no), visit dentist annually (yes/no), nutritional risk score, handgrip strength, and CRP.

Beside the cirrhosis (alcoholic cirrhosis, MELD score), nutritional (nutritional risk score, handgrip strength) and systemic inflammation parameters (CRP), the predictor variables were selected in advance from those revealed to have an association with periodontitis as described in previous studies [23]. Continuous variables were entered into the analyses in untransformed form.

A *P*- value of 0.05 or less was considered to be statistically significant. The data were analyzed using Stata version 12.0 (Stata Corp LP, College Station, TX).

## Results

**Fig. 1:**
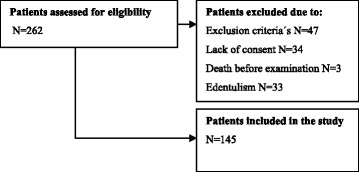
Patient inclusion flowchart

We screened 262 patients for eligibility, of which 117 were excluded by the exclusion criteria, lack of consent, death before the oral examination, or edentulism. Figure 1 gives the patient flow. A total of 145 patients were included into the study. Their mean age at baseline was 61 years (range 21–87 years), and 65% were men. Their clinical characteristics and demographic characteristics according to periodontitis status are presented in Table 2.

**Table 2:** Characteristics of the patient cohort

	No-or-mild periodontitis	Moderate periodontitis	Severe periodontitis
Number of patients	22	57	66
Age	57 (44–67)	61 (57–67)	62 (58–69)
Female / Male (%)	45 / 55	42 / 58	29 / 71
Cirrhosis etiology (%)			
Alcohol	55	72	83
Cryptogenic	5	14	12
Autoimmune or cholestastic	35	12	5
Viral B and / or C	5	2	
Cirrhosis severity^a^			
Model of End-Stage Liver Disease score	10 (6–15)	12 (8–17)	11 (6–14)
Smoker status (%)			
Yes/No	32 / 68	37 / 63	51 / 49
Alcohol consumption (%)			
Yes / No	23 / 77	44 / 56	43 / 57
Charlson comobidity index (%)			
0	63	68	56
1	32	18	29
2	5	7	15
3+		7	
Oral care habits (%)			
Brush teeth twice daily	59	46	27
Visit dentist annually	45	23	29
Periodontal measures			
Number of teeth^a^	27 (21–29)	25 (10–27)	23 (16–27)
Sites with plaque (%)	55	75	83
Probing depth (mm)^a^	1.97 (1.62–2.14)	2.70 (2.28–3.04)	3.57 (3.28–3.94)
Clinical attachment level (mm)^a^	2.07 (1.79–2.42)	2.96 (2.54–3.51)	4.04 (3.65–4.65)
Sites with bleeding on probing (%)	18	41	64
Nutritional status^a^			
Nutritional risk score	3 (2–4)	3 (2–4)	4 (3–5)
Handgrip strength (kg)	32 (22–38)	25 (17–27)	22 (16–30)
Inflammation status^a^			
C-reactive protein (mg/L)	9.9 (4.6–25.2)	12.7 (7.2–29.0)	17.1 (5.8–36.6)

^a^median (interquartile range)

Forty-six percent of the patients had severe periodontitis, 39% had moderate periodontitis, and only 15% had no-or-mild periodontitis.

There were no differences in age, gender, cirrhosis etiology, cirrhosis severity, smoking status, alcohol use, comorbidity, dental visits, number of teeth, nutritional and inflammation status in patients with severe periodontitis as opposed to patients with no- or-mild and moderate periodontitis. However, patients with severe periodontitis brushed teeth less often, had more plaque, increased probing depth, increased clinical attachment level, and more bleeding on probing than patients without severe periodontitis (Table 2).

Smoking was a predictor of severe periodontitis (odds ratio (OR) 2.93, 95% confidence interval (CI): 1.29–6.63). The same was found for oral health markers in the form of brushing teeth twice daily (OR 0.30, 95% CI 0.11–0.79) and visiting the dentist annually (OR 3.51, 95% CI 1.22–10.81) (Table 3). Cirrhosis etiology and cirrhosis severity were not associated with severe periodontitis (Table 3).

**Table 3:** Logistic regression analyses of the association of the predictor variables with the outcome variable severe periodontitis

Variables	Univariable		Multivariable		Stepwise multivariable	
	Odds ratio	95% CI	Odds ratio	95% CI	Odds ratio	95% CI
Age, per year increase	1.02	0.99–1.06	1.02	0.98–1.06		
Male (yes/no)	1.87	0.93–3.74	1.59	0.66–3.85		
Alcoholic cirrhosis (yes/no)	2.27	0.97–5.07	1.65	0.58–4.71		
MELD score	0.97	0.92–1.02	0.93	0.87–1.01		
Smoking (yes/no)	2.05	1.04–4.05*	2.55	1.07–6.12*	2.93	1.29–6.63*
Alcohol use (yes/no)	1.24	0.63–2.41	0.68	0.27–1.73		
Diabetes (yes/no)	1.69	0.69–4.17	1.93	0.66–5.62		
Brush teeth twice daily (yes/no)	0.38	0.19–0.78*	0.33	0.13–0.91*	0.30	0.11–0.79*
Visit dentist yearly (yes/no)	0.98	0.48–2.03	3.51	1.17–10.52*	3.51	1.22–10.81*
Nutritional risk score	1.34	1.02–1.76*	1.57	1.11–2.24*	1.57	1.14–2.18*
Handgrip strength, per kg increase	0.94	0.87–1.02	0.98	0.82–1.16		
CRP, per mg/L increase	1.00	0.99–1.02	1.00	0.99–1.02		

*CI* confidence interval; *MELD score* model of end-stage liver disease; *CRP* C-reactive protein

*P < 0.05 group with severe periodontitis vs. no-mild, or moderate periodontitis

Compared to patients with no-or-mild and moderate periodontitis, patients with severe periodontitis had a higher nutritional risk score (3, interquartile range (IQR) 3–5 vs. 3, IQR 2–4) and lower handgrip strength (22 kg, IQR 16–30 kg vs. 25 kg, IQR 18–35 kg), but only the nutritional risk score was significantly different (*P* = 0.02 and *P* = 0.1). Likewise, the median CRP concentration was higher in patients with severe periodontitis than in those without (17.1 mg/L, IQR 5.8–36.6 mg/L vs. 12.4 mg/L, IQR 6.7–27.1 mg/L), but there was no statistically significant difference (*P* = 0.08) (Table 3).

**Fig. 2:**
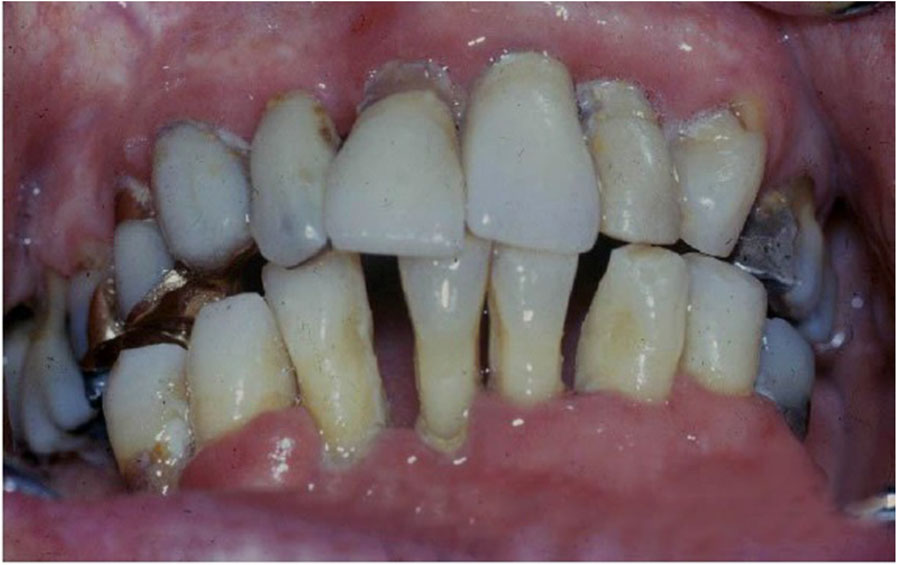
Severe periodontitis in a patient

Figure 2 shows a photograph of severe periodontitis.

## Discussion

This study describes a very high, close to universal, prevalence of periodontitis in patients with cirrhosis independent of cirrhosis etiology and severity. Within the patient population smoking and oral care habits were identified as predictors of periodontitis. In addition, it is the first study to suggest a relationship between periodontitis and nutritional status in cirrhosis patients.

Previous authors have recorded periodontal disease in 32–52% of their cirrhosis cohorts [5, 8] but unfortunately, a direct comparison is difficult because periodontitis is defined by different criteria. No definition, measures, or indices have gained universal approval [24]. In this study we used the definition proposed by the CDC/AAP [17]. This definition is becoming more widely used making comparisons between our and future studies possible [25].

In this study, all of the periodontal disease indicators (plaque, BOP, PD, CAL) were involved, indicating full clinical penetrance of periodontitis. Diagnostic uncertainty notwithstanding, the prevalence of periodontitis in this study population was markedly higher than the 10–15% in the adult general population worldwide [26, 27]. Thus, the improvement in the general population’s oral health during the past decade [28] is not followed by a similar improvement in patients with cirrhosis.

Alcoholic cirrhosis was not associated with severe periodontitis which is surprising as alcoholic cirrhosis is often linked to a lifestyle with poor self-care, resulting in poor oral health and increased oral infections [7, 9]. However, our finding is in accordance with others [8]. The explanation may be a consequence of the immune dysfunction that occurs progressively during the course of cirrhosis leading to an increased susceptibility to bacterial infections in the patients [29], and an increased risk of periodontitis. Another putative mechanism could be a reduced saliva flow rate. A number of factors associated with cirrhosis, such as the pharmacological management of ascites, can diminish the amount of saliva, which in turn, increases the development of plaque and favours oral infections [30, 31]. Furthermore, autoimmune hepatitis, and primary biliary cirrhosis have been associated with Sjögren’s syndrome, another cause of oral dryness [32].

Likewise, cirrhosis severity measured by the MELD score was not a predictor for severe periodontitis, although another study has reported an association between oral infections and accelerated progression of liver disease measured by the MELD score [33]. Thus, our findings indicate that it is the cirrhosis and the associated poor oral health status rather than the etiology or severity of cirrhosis which predisposes to severe periodontitis.

The results showed that smoking and brushing teeth less than twice daily were associated with severe periodontitis in the patients with cirrhosis. The importance of these factors is well known and documented in the general population [23, 34]. However, the observation that severe periodontitis was more prevalent among those visiting the dentist annually was unexpected and not explainable by our data. It may indicate either that dentists in Denmark do not treat severe periodontitis, that cirrhosis patients with severe periodontitis seek treatment more often than patients with good periodontal status, or that patients with few dental visits tend to have teeth with severe periodontitis extracted rather than treated.

The study suggested that severe periodontitis in cirrhosis patients was associated with the robust nutritional marker the nutritional risk score. This has not previously been studied in cirrhosis but is consistent with haemodialysis patients, where a relationship between malnutrition and periodontitis have been found [14, 35]. The majority of our patients suffered from severe dys-nutrition which is a massive and partly unexplained complication to cirrhosis that contributes to increased morbidity and mortality [12]. If a causal relationship between periodontitis and the degree of dys-nutrition can be established by future studies the care for cirrhosis patients should probably involve periodontal treatment. The mechanism of an effect of periodontitis on nutrition cannot be established from our data, but probably both unpleasant oral eating related experiences, such as earlier described [3], and the anorectic effect of the low grade systemic inflammation activation reported to stem from periodontitis may contribute.

CRP is a sensitive marker of systemic inflammation, and periodontitis may be associated with an increase in CRP levels [36]. Our cirrhosis cohort had increased CRP, to a large extent attributable to cirrhosis as a chronic inflammatory condition. On this platform we found only a slight stepwise increase in CRP with the severity of periodontitis but not significant. This may imply that the value of CRP for detecting systemic inflammation from periodontitis is weak in patients with cirrhosis [37].

There are limitations to this study. The study design provided only association among the study variables and not on causality. Therefore, further research must be done before the potential for oral infections to cause damage in cirrhosis patients can be definitely established. In particular, longitudinal studies with repeated oral examinations and oral interventions studies would be valuable. In addition, the findings could to some extent be due to confounding variables, such as unmeasured socioeconomic conditions, as it is shown that lower socioeconomic groups have lack of oral health awareness and dental care [38]. The possible confounding by these conditions is worth investigating in future studies. Finally, our study comprised a broad spectrum of cirrhosis patients and may be seen as representative for a cirrhosis population, but still the study design may limit generalizability of the strength of the association to other patients with cirrhosis. However, in this case, this seems unlikely as the department has a large local catchment population and also receives referred patients from other hospitals across Denmark. In addition, age, gender, cirrhosis etiology, cirrhosis severity, and comorbidities of the participating patients correspond to Danish nationwide cohort studies and other Scandinavian cohort studies performed in the recent years [39–41]. However, we believe this study contributes with new information in the field of oral health and infections in patients with cirrhosis.

## Conclusion

In conclusion, it was observed that periodontitis is highly prevalent in cirrhosis patients of all etiologies. Further predictors of severe periodontitis were smoking, brushing teeth less than twice daily and, unexpectedly visiting the dentist annually. Importantly, severe periodontitis was associated with a high nutritional risk score. These findings motivate further studies including interventional trials to evaluate whether improved clinical care of oral health may improve the nutritional status in patients with cirrhosis.

## Abbreviations

AAP: American Academy of Periodontology
BOP: bleeding on probing
CAL: clinical attachment level
CDC: Centre for Disease Control
CI: confidence interval
CRP: C-reactive protein
IQR: interquartile range
MELD: Model of End-Stage Liver Disease
OR: odds ratio
PD: probing depths
